# Antagonistic Transcriptional and Metabolic Networks Are Associated with Anthocyanin Accumulation in Maize Seedlings

**DOI:** 10.3390/genes17080866

**Published:** 2026-07-24

**Authors:** Yuan Ren, Junwen Meng, Jin Zhang, Qijian Tian, Rui Huang, Xin Liu

**Affiliations:** 1 Center for Agricultural Genetic Resources Research, Shanxi Agricultural University, Taiyuan 030031, China; ryuan007@sxau.edu.cn (Y.R.); sxmjw@163.com (J.M.); zhangjin2020@sxau.edu.cn (J.Z.); pzstqj2008@163.com (Q.T.); huangrui@sxau.edu.cn (R.H.); 2 Shanxi Functional Food Research Institute, Shanxi Agricultural University, Taiyuan 030031, China

**Keywords:** anthocyanin, maize, metabolomics, transcriptomics, WGCNA, MYB transcription factors

## Abstract

**Background:** Anthocyanin accumulation is a developmentally regulated trait shaped by complex interactions between metabolic and transcriptional networks. However, dissecting the regulatory mechanisms underlying anthocyanin biosynthesis is often complicated by confounding variation in plant growth and environmental conditions. **Methods:** Here, we used a time-resolved multi-omics approach to investigate anthocyanin accumulation in maize seedlings by comparing an anthocyanin-rich inbred line PH19401 with an anthocyanin-deficient line YPX across five developmental stages. **Results:** Untargeted metabolomic and transcriptomic profiling revealed progressive divergence between the two lines beginning at early development stages. Using a dual-line, intersection-based filtering strategy, we identified a refined set of metabolites and genes closely associated with anthocyanin accumulation. These candidates were enriched in pathways related to phenylpropanoid metabolism, energy metabolism, and redox regulation. Weighted gene co-expression network analysis (WGCNA) identified two transcriptional modules that showed opposing associations with anthocyanin content. The positively associated module was centered on MYB transcription factors, consistent with canonical regulation of flavonoid biosynthesis, whereas the negatively associated module was enriched in genes involved in primary metabolism and signaling. Integration of transcriptomic and metabolomic datasets further revealed coordinated relationships between MYB hub genes and flavonoid intermediates, linking transcriptional regulation with metabolic output. Together, these results support a model in which anthocyanin accumulation is associated with activation of MYB-centered transcriptional programs and broader metabolic reprogramming that enhances precursor supply and redox balance, while competing transcriptional programs favor primary metabolism. **Conclusions:** This study provides a systems-level perspective on anthocyanin biosynthesis in maize seedlings and establishes an analytical framework for dissecting developmentally regulated metabolic traits.

## 1. Introduction

Anthocyanins are a major class of flavonoid pigments that confer red, purple, and blue coloration to plant tissues and play critical roles in plant development, stress adaptation, and human nutrition [1,2,3]. In maize (*Zea mays* L.), anthocyanin accumulation not only determines visible pigmentation in leaves, stems, and kernels but also serves as a useful phenotypic marker in breeding programs and a functional indicator of metabolic and transcriptional reprogramming [4,5]. Beyond their aesthetic and agronomic value, anthocyanins contribute to photoprotection, antioxidant capacity, and tolerance to multiple abiotic and biotic stresses, highlighting their significance in both plant physiology and crop improvement [6,7,8]. Anthocyanin biosynthesis originates from the phenylpropanoid pathway and involves a series of well-characterized enzymatic steps. At the transcriptional level, this pathway is primarily regulated by the MYB-bHLH-WD40 (MBW) transcriptional complex, with additional modulation by light-responsive factors and hormone- and stress-related signaling pathways [9]. Although the core biosynthetic and regulatory components have been extensively studied, anthocyanin accumulation in planta is increasingly recognized as a coordinated outcome of metabolic flux, precursor availability, redox balance, and transcriptional network regulation [10,11].

Despite substantial progress, a major challenge in dissecting anthocyanin regulation is distinguishing anthocyanin-specific regulatory signals from broader transcriptional and metabolic changes associated with plant development. Anthocyanin accumulation is often developmentally regulated and emerges gradually across growth stages and tissues [12]. Consequently, conventional single-time-point comparisons or simple pairwise contrasts may capture a mixture of pigmentation-related signals and background variation driven by developmental progression, environmental fluctuations, or experimental conditions [13]. This confounding effect limits the ability to identify core regulators and metabolites specifically associated with anthocyanin biosynthesis.

Recent advances in high-throughput transcriptomics and metabolomics have enabled integrative multi-omics approaches to investigate secondary metabolism at increasing resolution [14,15]. In particular, time-series profiling combined with network-based analyses, such as weighted gene co-expression network analysis (WGCNA), provides a powerful framework for identifying regulatory modules and hub genes underlying complex traits [16]. However, many existing multi-omics studies of anthocyanin biosynthesis rely on single genotypes or lack explicit strategies to reduce developmental background noise, which may lead to inflated candidate lists and reduced interpretability [17].

In this study, we employed a time-resolved, dual-line multi-omics strategy to systematically dissect the regulatory framework underlying anthocyanin accumulation in maize seedlings. We analyzed an anthocyanin-rich inbred line, PH19401, and an anthocyanin-deficient line, YPX, across five developmental stages spanning the onset and progression of pigmentation. By integrating untargeted metabolomics and transcriptomics, we implemented an intersection-based filtering strategy designed to progressively eliminate nonspecific developmental effects and enrich for anthocyanin-associated metabolites and genes. We then used WGCNA to identify co-expression modules and hub regulators correlated with anthocyanin accumulation, followed by gene-metabolite correlation analysis to link transcriptional regulation with metabolic output. Through this integrative approach, we reveal a coordinated regulatory framework associated with anthocyanin accumulation, involving both canonical biosynthetic regulators and antagonistic transcriptional modules linked to metabolic and redox processes. These findings provide a systems-level perspective on anthocyanin biosynthesis in maize and offer a robust analytical framework applicable to other developmentally regulated metabolic traits.

## 2. Materials and Methods

### 2.1. Plant Materials and Growth Conditions

The anthocyanin-rich maize inbred line PH19401 and the anthocyanin-deficient inbred line YPX were used in this study. PH19401 was derived from a cross between a Peruvian black maize landrace and the commercial hybrid Xianyu 335, followed by inbreeding and selection for high anthocyanin accumulation. YPX was developed in our laboratory by crossing Zheng 58 with HDP933, followed by seven generations of self-pollination, and was selected for the complete absence of anthocyanin pigmentation in all tissues. Seeds were surface-sterilized with 10% hydrogen peroxide, rinsed thoroughly three times with deionized water, and germinated at 25 °C for 3 days. Uniform seedlings were then transferred to growth chambers (RXZ, Ningbo Southeast Instrument Co., Ltd., Ningbo, China) maintained at 25 °C and 60% relative humidity under a 16-h light/8-h dark photoperiod. Leaf samples were collected at 10, 20, 30, 40, and 50 days after seedling emergence, corresponding to stages S1–S5. Samples from PH19401 and YPX were designated PH_S1-PH_S5 and YP_S1-YP_S5, respectively. All samples were immediately snap-frozen in liquid nitrogen and stored at −80 °C until subsequent analysis.

### 2.2. Reagents

All chemicals were of analytical grade or higher. HPLC-grade methanol, acetonitrile, and formic acid, as well as TRIzol reagent and the NEBNext Ultra RNA Library Prep Kit, were obtained from commercial suppliers. The pH differential assay buffers (pH 1.0 and pH 4.5) were prepared in-house using ultrapure water. Deionized water (resistivity ≥ 18.2 MΩ·cm) was used for all aqueous solutions.

### 2.3. Quantification of Total Anthocyanin Content in Maize Seedlings

Total anthocyanin content was determined using the pH differential method. Fresh maize tissue was ground in liquid nitrogen, and 0.1 g of powdered sample was extracted with 1 mL of acidified methanol containing 1% HCl. The mixture was incubated at 4 °C in the dark for 12–16 h with gentle shaking and then centrifuged at 12,000× *g* for 10 min at 4 °C. The supernatant was collected and diluted appropriately using two buffer systems: 0.025 M potassium chloride buffer, pH 1.0, and 0.4 M sodium acetate buffer, pH 4.5. After equilibration for 15 min at room temperature in the dark, absorbance was measured at 510 and 700 nm using a UV-visible spectrophotometer (TU-1900, Beijing Purkinje General Instrument Co., Ltd., Beijing, China).

### 2.4. Metabolite Extraction and LC–MS/MS Analysis

For each genotype and developmental stage, four biological replicates were analyzed. Samples were preserved on dry ice and transported to NovoMagic for untargeted metabolomics analysis. Leaf tissues were vacuum freeze-dried and ground into a fine powder using a mixer mill (MM 400, Retsch, Retsch, Haan, Germany). For metabolite extraction, 0.1 g of powdered sample was extracted with 1 mL of 70% methanol and incubated overnight at 4 °C with intermittent vortexing to improve extraction efficiency. After centrifugation at 10,000× *g* for 10 min at 4 °C, the supernatants were collected and filtered through 0.22-μm organic microporous membranes before UPLC-MS/MS analysis [18].

Metabolite profiling was performed using an ultra-performance liquid chromatography system (Shim-pack UFLC SHIMADZU CBM30A, Shimadzu Corporation, Kyoto, Japan) coupled to a tandem mass spectrometry system (Applied Biosystems 6500 QTRAP, AB Sciex LLC, Framingham, MA, USA). Raw data were processed using SCIEX Analyst 1.6.3, and metabolites were identified by matching accurate mass, retention time, and fragmentation spectra against the Novogene in-house database, novoDB. Metabolite quantification was performed using the corresponding analytical software and the internal metabolite database. Multivariate analysis, including orthogonal partial least squares-discriminant analysis (OPLS-DA), was used to assess group separation and calculate variable importance in projection (VIP) values. Differentially accumulated metabolites were defined as those with VIP ≥ 1, fold change ≥2 or ≤0.5, and *p* < 0.05 based on one-way analysis of variance (ANOVA) [19].

### 2.5. RNA Sequencing and Transcriptomic Data Analysis

Total RNA was extracted from flash-frozen leaf samples using TRIzol reagent (Invitrogen, Carlsbad, CA, USA). RNA quality was assessed by agarose gel electrophoresis to evaluate degradation and contamination, a NanoPhotometer spectrophotometer (Implen GmbH, Munich, Germany) to determine purity, Qubit fluorometric quantification (Thermo Fisher Scientific, Waltham, MA, USA) to measure concentration, and an Agilent Bioanalyzer 2100 (Agilent Technologies, Santa Clara, CA, USA) to assess RNA integrity [20]. Only RNA samples with an RNA integrity number (RIN) ≥ 7.0 were used for library construction. Libraries were constructed from 3 µg of RNA per sample using the NEBNext Ultra RNA Library Prep Kit (New England Biolabs, Ipswich, MA, USA) for Illumina and quality-checked on an Agilent Bioanalyzer 2100 (Agilent Technologies, Santa Clara, CA, USA). Final libraries were clustered on a cBot system and sequenced on an Illumina HiSeq X platform by Novogene (Beijing, China) to generate 150-bp paired-end reads [21].

Clean reads were aligned to the maize reference genome B73_RefGen_v5 (https://www.maizegdb.org/; accessed on 20 April 2026) using HISAT2. Gene annotations were obtained from public databases, including the NCBI non-redundant protein sequence database (NR), Protein family database (Pfam), Clusters of Orthologous Groups/evolutionary genealogy of genes: Non-supervised Orthologous Groups databases (COG/KOG/eggNOG), Swiss-Prot, Kyoto Encyclopedia of Genes and Genomes (KEGG), and Gene Ontology (GO). Gene expression levels were calculated as fragments per kilobase of exon model per million mapped fragments (FPKM). Differentially expressed genes (DEGs) were identified using the DESeq2 R package with the criteria |log_2_ (fold change)| ≥ 1 and adjusted *p* value < 0.05. GO and KEGG pathway enrichment analyses were performed using OmicShare tools (https://www.omicshare.com/; accessed on 20 April 2026) [22].

### 2.6. Weighted Gene Co-Expression Network Analysis (WGCNA)

A weighted gene co-expression network was constructed using the WGCNA package in R to identify gene modules and hub genes associated with anthocyanin accumulation [23]. Genes with FPKM values greater than 1 across all samples were included in the analysis [24]. The soft-thresholding power was selected to approximate scale-free topology. A topological overlap matrix (TOM) was then calculated, and co-expression modules were identified using dynamic tree cutting. Correlations between module eigengenes and anthocyanin content in the two inbred lines, PH19401 and YPX, were calculated to identify trait-related modules. Hub genes within key modules were identified based on intramodular connectivity and betweenness centrality [25]. Networks were visualized using Cytoscape version 3.9.1, and graphical data were presented using the ggplot2 package in R.

### 2.7. Integrated Transcriptome-Metabolome Analysis

For each developmental stage and genotype, transcriptomic data, represented by FPKM values, and untargeted metabolomic data, represented by relative metabolite abundances, were obtained. Candidate genes of interest, including genes identified by KEGG enrichment analysis and hub genes identified by WGCNA, and core metabolites screened using the dual-line intersection strategy were selected for joint analysis. Pearson correlation coefficients were calculated between the expression level of each candidate gene and the relative abundance of each core metabolite using the combined dataset across all sample groups. Significant correlations were defined as |r| > 0.8 and *p* < 0.05. Gene-metabolite interaction networks were visualized using Cytoscape, with nodes representing genes or metabolites and edges representing significant positive or negative correlations.

### 2.8. Statistical Analysis

Statistical analyses were performed using SPSS version 22.0 (IBM Corp., Armonk, NY, USA). Differences among groups were evaluated by ANOVA followed by Fisher’s least significant difference (LSD) test, with statistical significance defined as *p* < 0.05. Unless otherwise specified, results are presented as the mean ± standard error (SE) based on three independent biological replicates. Data were visualized using Origin 2022 (OriginLab Corp., Northampton, MA, USA). WGCNA interaction networks and bubble plots were constructed and visualized using the OmicStudio online platform, and heatmaps of differentially accumulated metabolites were generated using TBtools version 2.0 [26].

## 3. Results

### 3.1. Developmental Divergence of Anthocyanin Accumulation Is Captured by Metabolomic Profiling

**Figure 1:**
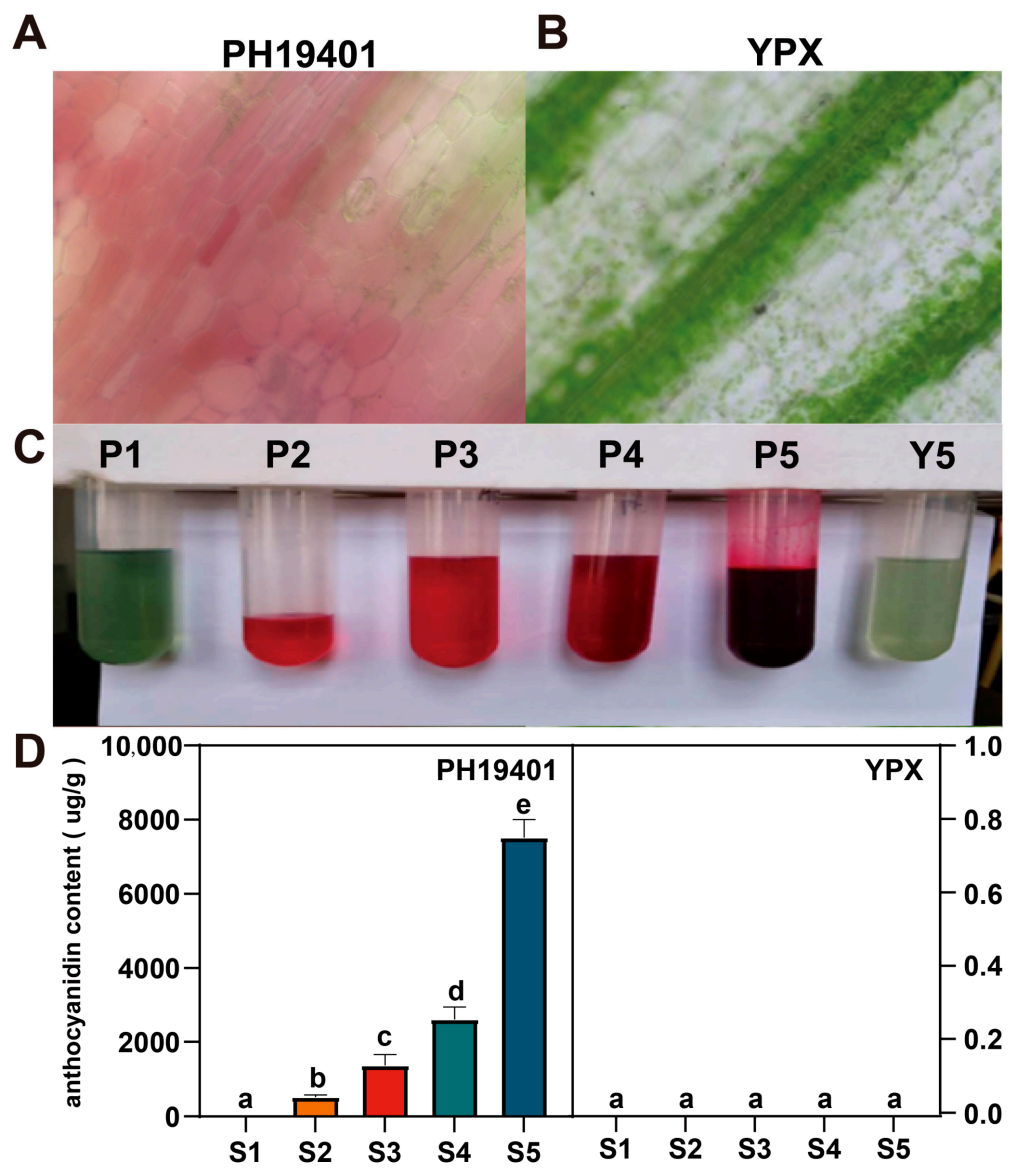
**Phenotypic comparison of anthocyanin accumulation between the anthocyanin-rich maize line PH19401 and the anthocyanin-deficient line YPX at different developmental stages.** (**A**,**B**) Microscopy images showing cellular-level differences of anthocyanin accumulation in PH19401 (P) and YPX (Y). (**C**) Visual comparison of anthocyanin extracts from leaf, showing clear differences in pigmentation between the two lines. samples were collected at 10, 20, 30, 40, and 50 days after seedling emergence, corresponding to stages S1–S5. (**D**) Quantitative analysis of total anthocyanin content in both lines at different developmental stages. Data are presented as means ± SD (n = 3). Different lowercase letters (a–e) indicate significant differences among developmental stages within each line, as determined by one-way ANOVA followed by post-hoc multiple comparisons (*p* < 0.05).

Anthocyanin accumulation in maize seedlings is a developmentally regulated process that emerges progressively and may be confounded by broader metabolic and transcriptional changes associated with plant growth. To distinguish anthocyanin-associated regulation from these developmental effects, we analyzed an anthocyanin-rich line PH19401 and an anthocyanin-deficient line YPX across a defined developmental time course. Microscopic examination revealed clear cellular differences in anthocyanin accumulation between the two lines (Figure 1A,B). Leaf samples were collected at 10, 20, 30, 40, and 50 days after seedling emergence, corresponding to developmental stages S1–S5, respectively. S1 represented the newly emerged seedling stage, whereas S2, S3, S4, and S5 corresponded to the three-leaf, six-leaf, ten-leaf, and flowering stages, respectively. In PH19401, visible pigmentation was absent at S1, became apparent at S2, and progressively intensified thereafter. By contrast, YPX remained non-pigmented throughout the developmental stages (Figure 1C). Quantification of total anthocyanin content confirmed the progressive increase in PH19401 and the absence of comparable anthocyanin accumulation in YPX (Figure 1D).

**Figure 2:**
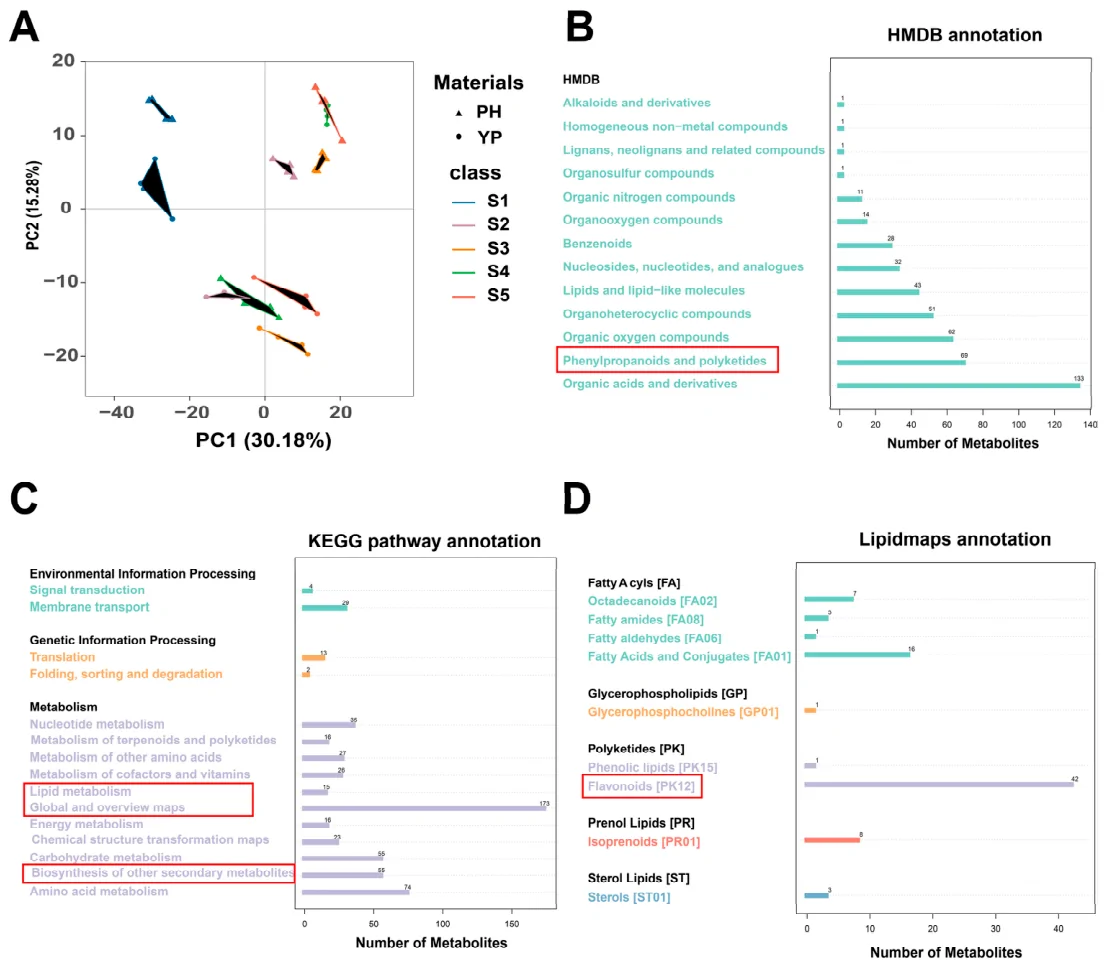
**Global metabolite profiling and functional annotation.** (**A**) Principal component analysis (PCA) plot of all samples based on untargeted metabolomic profiles, showing metabolic separation between the two lines and among developmental stages. (**B**) HMDB-based classification of detected metabolites, with organic acids and derivatives, phenylpropanoids and polyketides, organoheterocyclic compounds, and lipids representing the major metabolite classes. (**C**) KEGG-based classification of detected metabolites, showing prominent representation of metabolism-related pathways. (**D**) LipidMaps-based classification of detected metabolites, identifying polyketides, mainly flavonoids, as the most prominent lipid-related class.

To identify metabolites and genes associated with anthocyanin accumulation, we performed untargeted metabolomics using a dual-line, time-resolved filtering strategy. Principal component analysis (PCA) revealed minimal separation between PH19401 and YPX at S1, suggesting broadly similar metabolic profiles before the onset of visible pigmentation. In contrast, the two lines became clearly separated from S2 onward, with the divergence becoming progressively more pronounced at later developmental stages (Figure 2A). Within each genotype, samples followed a continuous trajectory in PCA space, indicating that metabolic variation was strongly structured by developmental progression. The detected metabolites were classified using multiple databases. HMDB-based classification showed that organic acids and derivatives, phenylpropanoids and polyketides, organoheterocyclic compounds, and lipids were the major metabolite classes. KEGG-based classification indicated that the detected metabolites were predominantly associated with metabolism-related pathways. LipidMaps-based classification further identified polyketides, mainly flavonoids, as the most prominent lipid-related class (Figure 2B–D; Tables S1 and S2). Together, these analyses generated a broad metabolite dataset encompassing diverse primary and secondary metabolic pathways.

### 3.2. A Dual-Line Filtering Strategy Identifies Anthocyanin-Associated Metabolites

**Figure 3:**
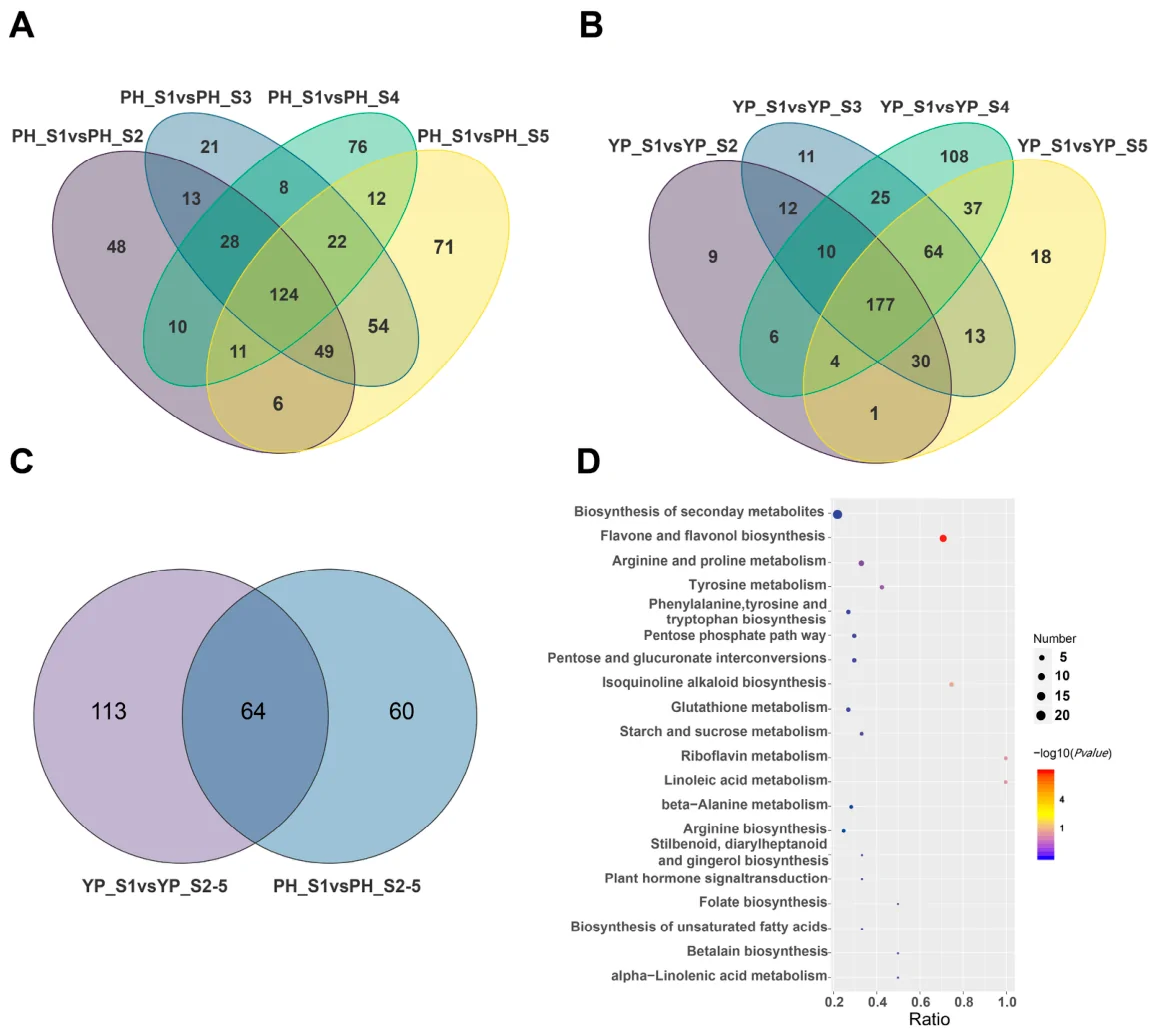
**Intersection-based filtering strategy for identifying core metabolites associated with anthocyanin accumulation.** (**A**) Venn diagram of differentially accumulated metabolites (DAMs) identified from four pairwise comparisons (PH_S1 vs. PH_S2, PH_S1 vs. PH_S3, PH_S1 vs. PH_S4, PH_S1 vs. PH_S5) in the anthocyanin-rich line PH19401. (**B**) Venn diagram of DAMs identified from the corresponding four pairwise comparisons in the anthocyanin-deficient line YPX. (**C**) Identification of candidate anthocyanin-associated core metabolites by excluding metabolites related to nonspecific developmental effects. (**D**) KEGG enrichment analysis of the 60 candidate metabolites obtained through intersection-based filtering.

To identify metabolites associated with anthocyanin accumulation while minimizing developmental background effects arising from developmental progression, we applied an intersection-based filtering strategy across developmental stages and genotypes. In PH19401, comparisons between S1 with each subsequent stages (S2–S5) identified 289–349 differentially accumulated metabolites per comparison, of which 124 were consistently detected across all four comparisons (Figure 3A; Table S3). These metabolites represented developmental metabolic changes in the anthocyanin-rich line but likely included both anthocyanin-related changes and broader developmental variation.

Applying the same analysis to YPX identified 177 metabolites that were consistently altered across developmental stages (Figure 3B), representing background developmental variation occurring in the absence of visible anthocyanin accumulation. After excluding metabolites shared between the two lines, we identified 60 metabolites that showed consistent developmental changes in PH19401 but not in YPX under the applied filtering criteria (Figure 3C). These metabolites were therefore considered high-confidence candidates associated with anthocyanin-related metabolic variation and were retained for subsequent analyses.

### 3.3. Anthocyanin-Associated Metabolites Are Enriched in Precursor and Flavonoid Pathways

To investigate the biological functions of the 60 candidate metabolites, we performed KEGG pathway enrichment analysis (Table S4). The enriched pathways were mainly associated with amino acid metabolism, energy metabolism, and plant secondary metabolism (Figure 3D). Notably, pathways associated with aromatic amino acid metabolism, including phenylalanine and tyrosine metabolism, were strongly enriched. Because aromatic amino acid metabolism is closely linked to the generation of precursors for phenylpropanoid and flavonoid biosynthesis, this enrichment suggests that changes in upstream metabolic processes may contribute to enhanced pigment accumulation. Consistent with this interpretation, pathways directly related to secondary metabolism, including flavonoid biosynthesis and broader secondary metabolite pathways, were also significantly enriched. In addition, the enrichment of cofactor- and energy-related pathways, such as aminoacyl-tRNA biosynthesis and glutathione metabolism, indicates that anthocyanin accumulation is accompanied by coordinated changes in cellular energy status and redox homeostasis. Together, these findings indicate that anthocyanin accumulation is possibly associated with coordinated remodeling of primary metabolism, precursor availability, redox regulation, and flavonoid biosynthesis.

### 3.4. Transcriptome Profiling Identifies Candidate Genes Associated with Anthocyanin Accumulation

**Figure 4:**
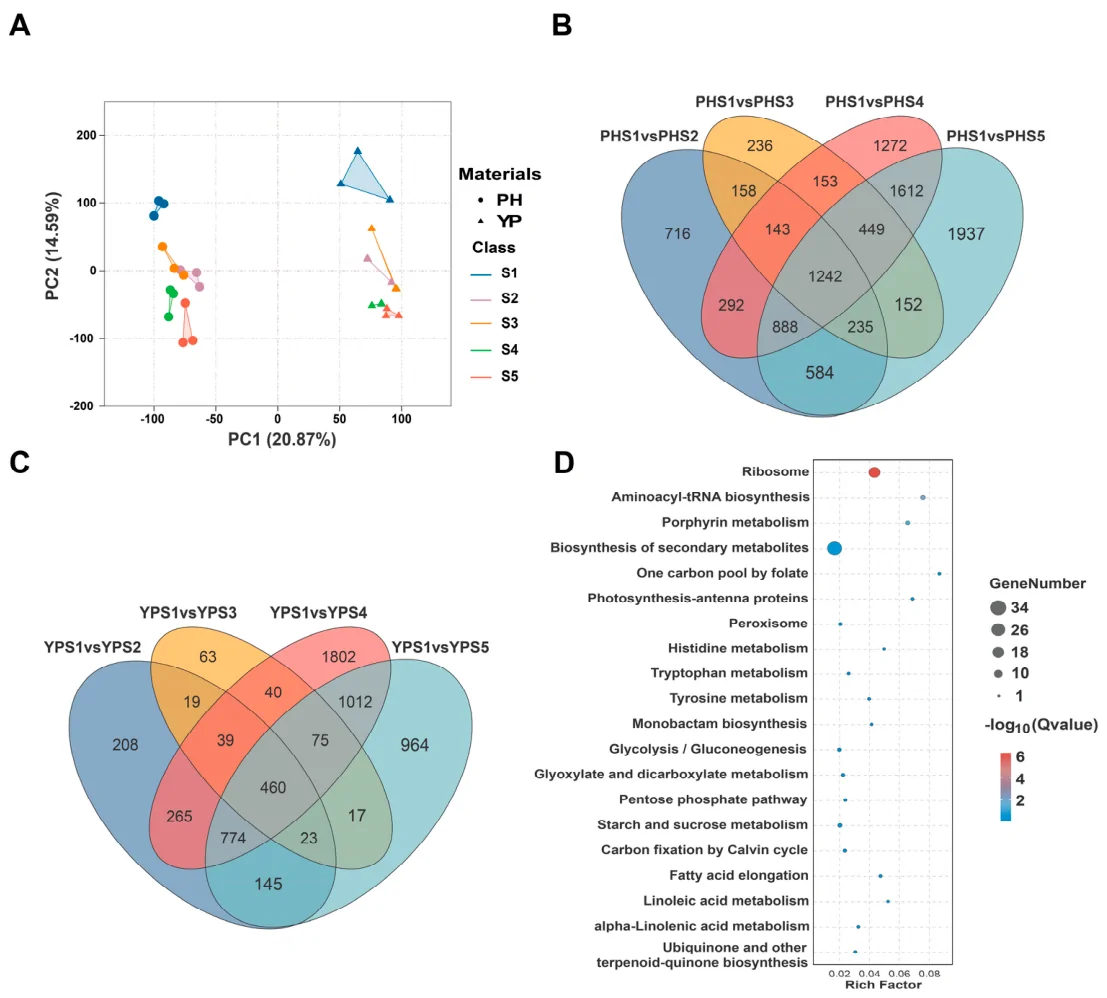
**Intersection-based filtering strategy for identifying candidate genes associated with anthocyanin accumulation.** (**A**) Principal component analysis (PCA) plot of all samples based on gene expression profiles, showing expression separation between the two lines and among developmental stages. (**B**) Venn diagram of differentially expressed genes (DEGs) identified from four pairwise comparisons (PH_S1 vs. PH_S2, PH_S1 vs. PH_S3, PH_S1 vs. PH_S4, PH_S1 vs. PH_S5) in the anthocyanin-rich line PH19401. (**C**) Venn diagram of DEGs identified from the corresponding four pairwise comparisons in the anthocyanin-deficient line YPX. (**D**) KEGG enrichment analysis of the 358 candidate genes obtained through intersection-based filtering.

To characterize the transcriptional dynamics underlying anthocyanin accumulation, we performed RNA-seq across both lines and developmental stages. High-quality sequencing data were obtained for all samples, with high read-mapping rates and strong consistency among biological replicates (Table S5). PCA of gene expression profiles revealed clear separation between PH19401 and YPX along the first principal component, indicating that genetic background was the dominant source of transcriptional variation, whereas the second principal component primarily reflected developmental progression (Figure 4A). Biological replicates clustered closely together, confirming the high reproducibility and reliability of the transcriptomic dataset.

Differential expression analysis using the same intersection-based strategy identified 1242 genes that were consistently altered across developmental stages in PH19401 (Figure 4B; Table S6). The corresponding analysis in YPX identified 460 consistently altered genes, which were considered to represent background developmental variation in gene expression (Figure 4C). After excluding genes shared between the two lines, 358 high-confidence candidate genes associated with anthocyanin-related transcriptional variation were retained. KEGG enrichment analysis of these genes revealed significant enrichment in pathways related to phenylalanine metabolism, the pentose phosphate pathway, and redox-associated processes (Figure 4D; Table S7). Among the 358 high-confidence candidate genes, three representative genes exhibiting the most significant differences of expression, *Zm00001d014659*, *Zm00001d053015*, and *Zm00001d027999*, showed stage-specific expression patterns and were therefore selected for further analysis. Functional annotation indicated that *Zm00001d014659* encodes a chemokine-like MDL protein, *Zm00001d053015* encodes a protein associated with tyrosine phosphorylation, and *Zm00001d027999* encodes a plastoquinone polyprenyltransferase. This refined candidate gene set provides a basis for subsequent network-based analyses aimed at elucidating regulatory relationships associated with anthocyanin accumulation.

### 3.5. Co-Expression Network Analysis Reveals Antagonistic Transcriptional Modules

**Figure 5:**
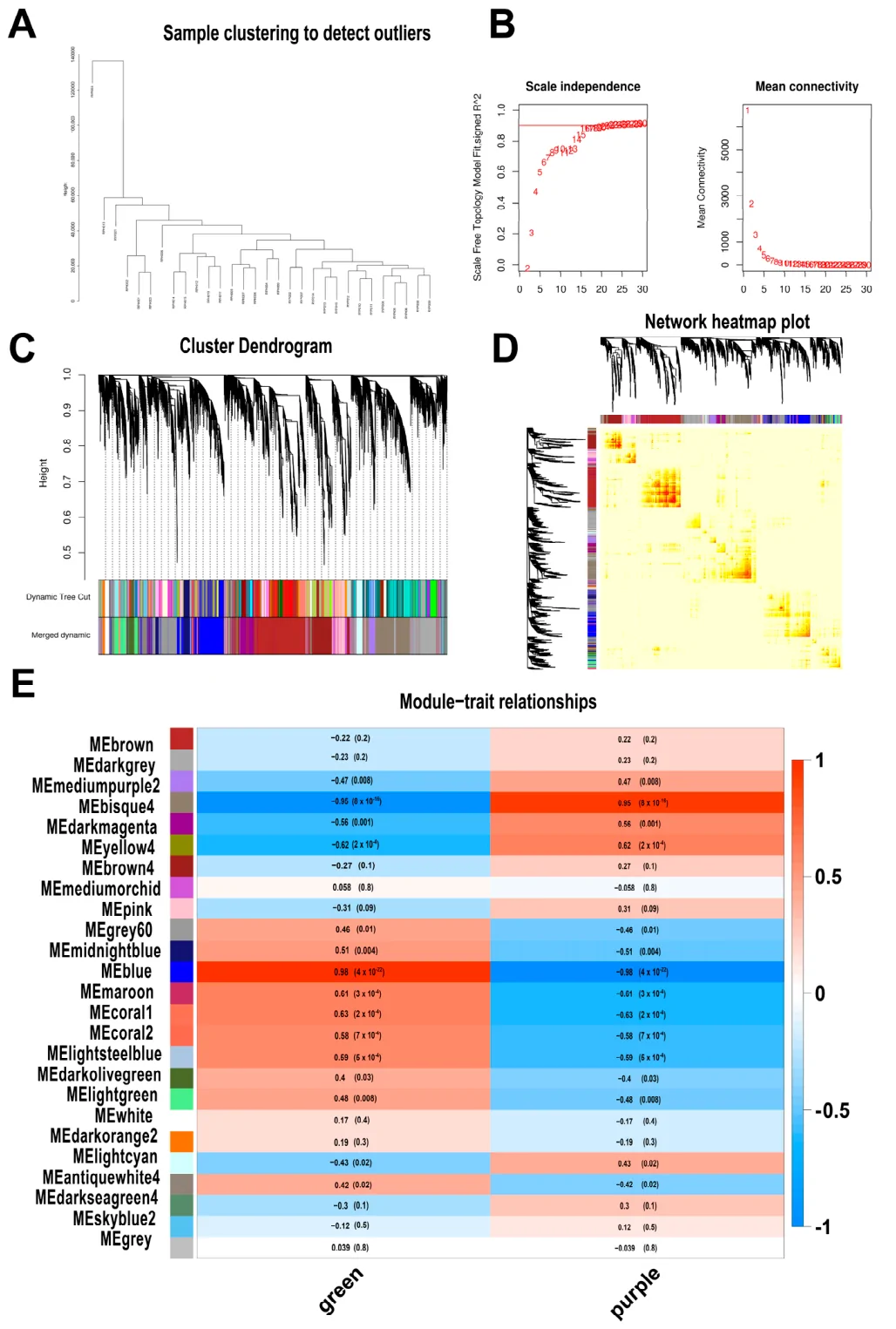
**Weighted gene co-expression network analysis (WGCNA) identifying modules associated with anthocyanin accumulation.** (**A**) Sample clustering dendrogram showing that no outlier samples were detected. (**B**) Network topology analysis for soft-thresholding power selection. A soft-thresholding power of β = 22 was selected, at which the scale-free topology fit index (R^2^) exceeded 0.85. (**C**) Hierarchical clustering dendrogram of genes, with module assignments indicated by color bars. (**D**) Network heatmap plot showing the number of genes assigned to each co-expression module. (**E**) Heatmap showing correlations between module eigengenes and anthocyanin-related traits in PH19401 and YPX.

WGCNA was performed to identify gene co-expression modules associated with anthocyanin accumulation in PH19401 and YPX. Sample clustering showed that all samples grouped appropriately, with no obvious outliers, indicating that the dataset was suitable for network construction (Figure 5A). Network topology analysis was then used to determine the optimal soft-thresholding power, and β = 22 was selected because the scale-free topology fit index exceeded 0.85 while maintaining relatively low mean connectivity (Figure 5B). Using this parameter, hierarchical clustering assigned the genes to 81 co-expression modules, as represented by distinct module colors (Figure 5C,D). Module-trait correlation analysis revealed that several modules were significantly associated with anthocyanin-related traits in PH19401 and YPX, with some displaying contrasting correlation patterns between the two genotypes (Figure 5E). In particular, the MEbisque4 module was strongly positively correlated with anthocyanin accumulation in the anthocyanin-rich line PH19401, whereas the MEblue module showed a stronger positive association with the anthocyanin-deficient phenotype of YPX. These results suggest that distinct gene co-expression networks may contribute to the differential regulation of anthocyanin accumulation in PH19401 and YPX.

**Figure 6:**
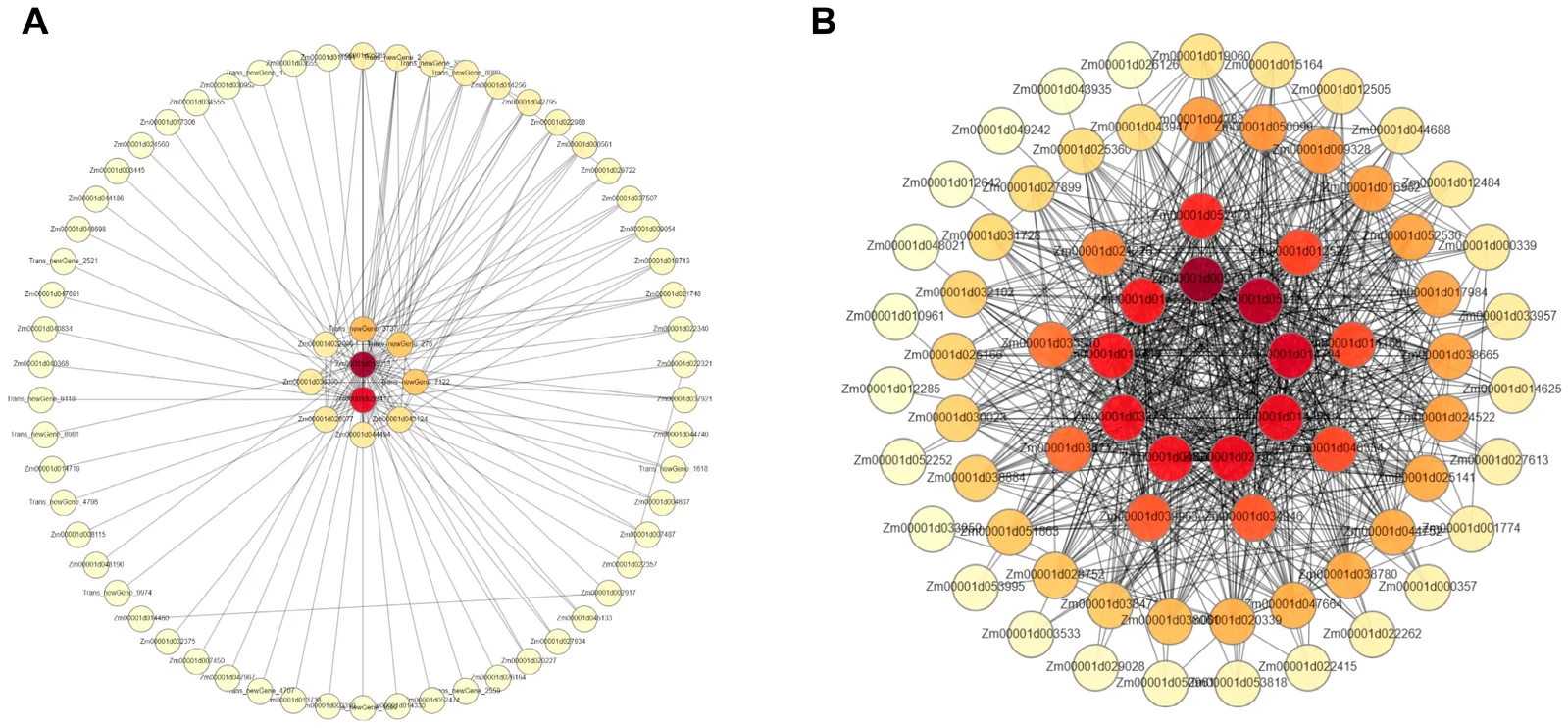
**Identification of hub genes within anthocyanin-associated co-expression modules.** (**A**) Subnetwork of the top 150 highly connected genes in the MEbisque4 module. Hub genes *Zm00001d018013* and *Zm00001d039417* were identified based on their top 5% rankings for both intramodular connectivity and betweenness centrality. (**B**) Subnetwork of the top 150 highly connected genes in the MEblue module. Nine hub genes were identified.

To identify potential key regulators within anthocyanin-associated co-expression modules, subnetworks were constructed using the top 150 most highly connected genes from the MEbisque4 and MEblue modules. The MEbisque4 subnetwork showed a relatively simple interaction structure, and two genes, *Zm00001d018013* and *Zm00001d039417*, were identified as hub genes because they ranked within the top 5% for both intramodular connectivity and betweenness centrality (Figure 6A). In contrast, the MEblue module formed a denser subnetwork, indicating more extensive co-expression relationships among its constituent genes. Nine hub genes, *Zm00001d009700*, *Zm00001d016719*, *Zm00001d053181*, *Zm00001d014794*, *Zm00001d014253*, *Zm00001d027932*, *Zm00001d048263*, *Zm00001d032753*, and *Zm00001d010788*, were identified in this subnetwork, suggesting that the MEblue module contains multiple highly connected genes potentially associated with differential anthocyanin accumulation (Figure 6B). Together with the three genes selected on the basis of their pronounced differential expression and stage-specific expression patterns, these 11 hub genes constituted a set of 14 high-confidence candidate genes for subsequent integrative analysis and future functional validation of the molecular processes associated with differential anthocyanin accumulation in PH19401 and YPX.

### 3.6. Integration of Transcriptome and Metabolome Defines a MYB-Centered Regulatory Network

**Figure 7:**
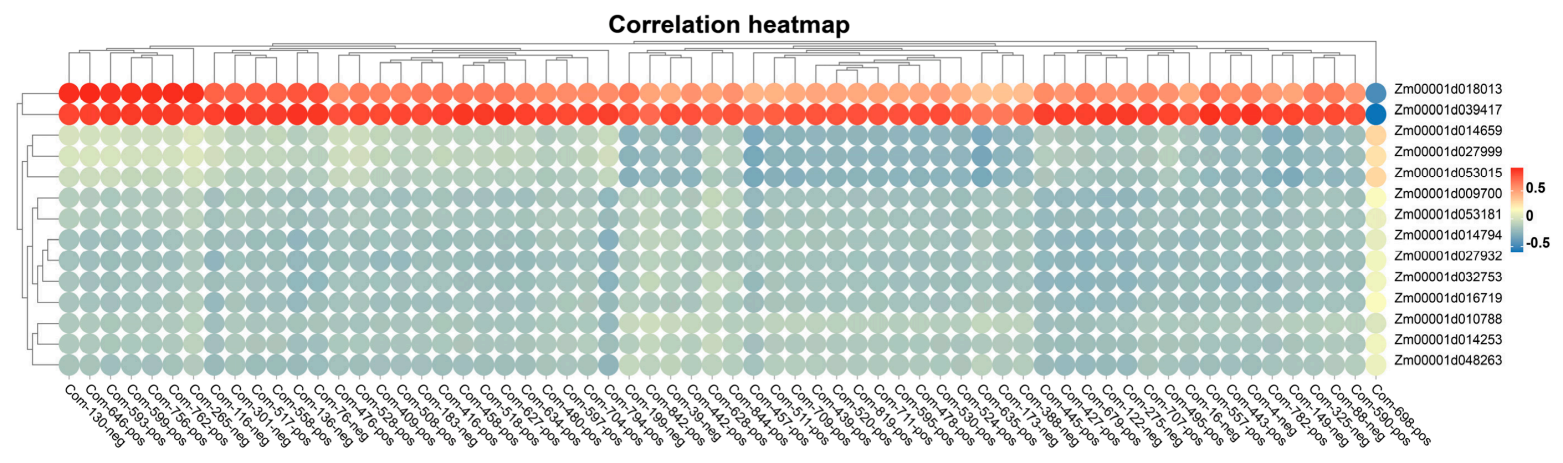
**Correlation analysis integrating 14 candidate genes with anthocyanin-associated core metabolites.** Heatmap showing Pearson correlation coefficients between 14 candidate genes and 60 anthocyanin-associated core metabolites.

To further evaluate the potential relationships between candidate genes and anthocyanin metabolism, Pearson correlation analysis was performed between the 14 candidate genes and 60 anthocyanin-associated core metabolites (Tables S8 and S9). The resulting heatmap revealed distinct correlation patterns, indicating that individual genes differed in their associations with specific metabolites (Figure 7). Several candidate genes showed broadly positive correlations with most core metabolites, suggesting that they may promote or coordinate anthocyanin accumulation. In contrast, other genes exhibited weaker or negative correlations with metabolite abundance, suggesting potentially distinct roles or associations within the underlying regulatory network. The two MYB transcription factors from the MEbisque4 module showed strong positive correlations with most metabolites, particularly those in the phenylpropanoid and flavonoid pathways, including key intermediates such as naringenin, dihydrokaempferol, and cyanidin derivatives. These relationships support their candidacy as central positive regulators of anthocyanin biosynthesis. In contrast, hub genes from the MEblue module were predominantly negatively correlated with these metabolites, suggesting that they may be associated with a competing regulatory program linked to reduced anthocyanin accumulation.

**Figure 8:**
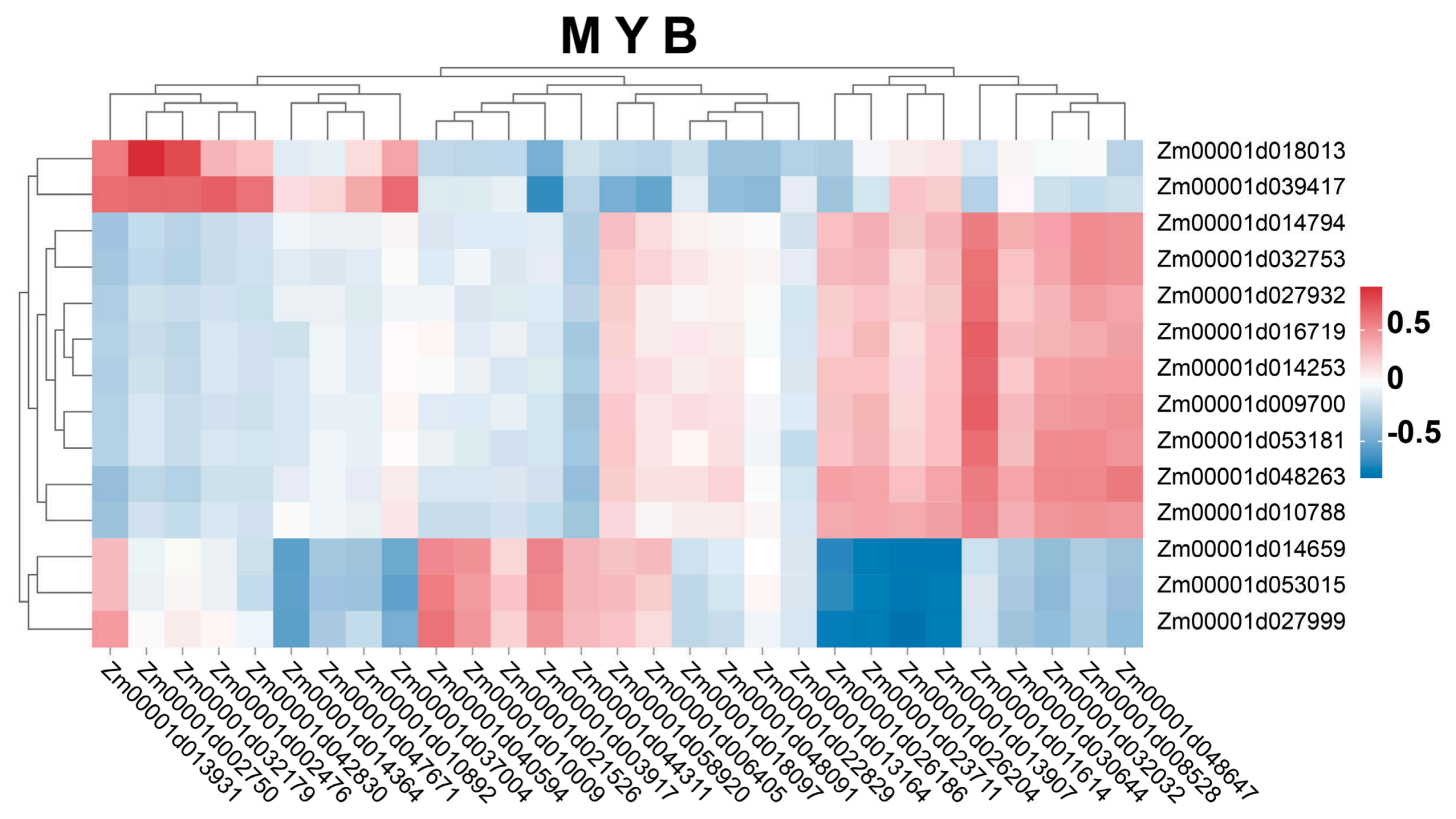
**Co-expression analysis of hub genes and MYB family transcription factors.** Heatmap showing Pearson correlation coefficients between the expression profiles of 14 candidate genes and all annotated MYB family genes.

To explore potential regulatory relationships between the identified candidate genes and MYB transcription factors, Pearson correlation analysis was performed using the expression profiles of the 14 candidate genes and all annotated MYB family genes (Figure 8, Table S10). The presence of distinct positively and negatively correlated MYB clusters suggests that different MYB transcription factors may have divergent or opposing associations with anthocyanin-related gene networks. Together, these results support a regulatory framework in which anthocyanin accumulation is associated with a MYB-centered transcriptional module linked to flavonoid biosynthesis, whereas an opposing module associated with primary metabolism may be linked to reduced anthocyanin accumulation.

## 4. Discussion

### 4.1. A Dual-Line, Time-Resolved Framework Refines the Identification of Anthocyanin-Associated Features

Anthocyanin accumulation is tightly coupled to developmental progression, making it challenging to distinguish pigmentation-specific regulation from broader physiological changes [27,28]. In this study, we addressed this challenge using a comparative, time-resolved multi-omics design incorporating both an anthocyanin-rich and an anthocyanin-deficient maize line. By comparing developmental changes within each line and removing shared signals between genotypes, we refined a set of metabolites and genes consistently associated with anthocyanin accumulation. For instance, integrated metabolomic and transcriptomic analyses of potato leaves, combined with *StAN1* overexpression, identified anthocyanin regulatory networks and demonstrate the utility of integrative approaches for resolving pigmentation-associated pathways [29]. Compared with previous studies that typically analyzed a single genotype or developmental stage, this approach provides more stringent control of developmental background effects and reduces the inclusion of indirect or nonspecific responses. The recovery of flavonoid-related metabolites and transcriptional regulators within the filtered dataset supports the biological relevance of this strategy. Rather than representing a conceptual shift, this framework provides a practical approach for improving signal specificity in studies of developmentally regulated metabolic traits. However, the observed divergence (Figure 2 and Figure 4A) may partly stem from pleiotropic growth differences between the two lines, underscoring the need for future studies incorporating broader genetic backgrounds and growth phenotyping to disentangle these confounders.

### 4.2. Anthocyanin Accumulation Is Associated with Coordinated Changes in Precursor Supply, Energy Metabolism, and Redox Balance

Metabolic analyses revealed that anthocyanin accumulation was associated with broad remodeling of primary metabolism, including pathways related to aromatic amino acid biosynthesis, energy metabolism, and redox homeostasis. The enrichment of phenylalanine and tyrosine metabolism is consistent with their established roles as precursors for phenylpropanoid and flavonoid biosynthesis. In addition, the enrichment of pathways such as the pentose phosphate pathway and glutathione metabolism suggests that anthocyanin accumulation is associated with increased reducing capacity and altered redox homeostasis. Anthocyanin biosynthesis requires reducing power and is often linked to cellular responses to oxidative conditions, further supporting this interpretation. The regulation of metabolic flux through the flavonoid pathway involves auxiliary proteins that channel intermediates toward specific branches, including anthocyanin biosynthesis [30]. Moreover, coordinated transcriptional reprogramming of primary and secondary metabolism can shift carbon allocation toward specialized metabolite production, as demonstrated in Medicago truncatula [31]. These findings are consistent with previous studies showing that anthocyanin production is integrated with broader metabolic and environmental responses rather than operating as an isolated biosynthetic process [32,33]. Together, these results indicate that anthocyanin accumulation is associated with coordinated metabolic reprogramming that supports precursor availability, energy supply, and redox balance. Notably, among the 358 candidate genes, those encoding enzymes at key nodes of the phenylpropanoid and flavonoid pathways represent the most directly anthocyanin-relevant components, whereas genes involved in energy and redox metabolism likely play supporting roles in precursor supply and cellular redox balance rather than serving as core biosynthetic constituents.

### 4.3. Antagonistic Transcriptional Modules Are Associated with Pigmented and Non-Pigmented States

Co-expression network analysis identified two modules with opposing relationships to anthocyanin accumulation (Figure 5E). The MEbisque4 module was positively associated with pigmentation and was centered on MYB transcription factors, whereas the MEblue module was associated with the non-pigmented state and enriched for genes involved in primary metabolism and signaling. Notably, the integration of hormonal signals with MBW complex activity provides an additional regulatory layer: DELLA proteins, key repressors of gibberellin signaling, promote anthocyanin biosynthesis by sequestering MYBL2 and JAZ repressors of the MBW complex, thereby linking growth-related hormonal pathways with anthocyanin accumulation [34]. The identification of MYB transcription factors as central hub genes is consistent with the well-established MYB-bHLH-WD40 (MBW) regulatory complex, which controls anthocyanin biosynthesis across plant species [35]. MYB components of this complex act as key determinants of pathway activation by coordinating the expression of structural genes involved in flavonoid biosynthesis.

In contrast, the MEblue module may reflect a transcriptional state associated with continued investment in primary metabolism and growth rather than secondary metabolite production. The negative association between this module and anthocyanin accumulation suggests that pigmentation may arise from a balance between competing transcriptional programs rather than activation of a single pathway alone. Similar trade-offs between growth and secondary metabolism have been reported in other plant systems [36]. The negative association between MEblue and anthocyanin accumulation permits two non-mutually exclusive interpretations. One possibility is true regulatory antagonism, whereby MYB-centered regulatory programs actively suppress primary metabolism and redirect resources toward flavonoid biosynthesis. Alternatively, the association may reflect a passive resource-allocation trade-off, in which the substantial metabolic cost of anthocyanin production indirectly constrains primary metabolism. Distinguishing between these scenarios will require metabolic flux analyses and genetic perturbation studies. Moreover, the phenotypic differences between the two lines may partly reflect pleiotropic effects on plant growth rather than differences in anthocyanin accumulation alone. This antagonistic framework is consistent with the broader understanding that secondary metabolism is regulated in coordination with growth and environmental conditions, allowing plants to balance resource allocation between primary and specialized metabolic processes.

### 4.4. Integration of Transcriptome and Metabolome Data Links Transcriptional Programs to Metabolic Output

Integration of gene expression and metabolite profiles revealed strong associations between MYB hub genes and flavonoid-related metabolites (Figure 7), including key intermediates such as naringenin and cyanidin derivatives. These correlations support the role of MYB-centered transcriptional programs in coordinating anthocyanin biosynthesis at the metabolic level. Conversely, hub genes from the MEblue module showed predominantly negative associations with these metabolites, reinforcing the presence of a competing transcriptional program. This integrated analysis connects transcriptional regulation with metabolic output and highlights candidate gene-metabolite relationships that can be tested in future functional studies. Together, these results support a model in which anthocyanin accumulation is associated with activation of MYB-centered transcriptional programs and broader metabolic changes that enhance precursor supply and redox balance, while alternative transcriptional programs favor primary metabolism.

### 4.5. Limitations and Future Directions

Several limitations should be considered when interpreting our findings. First, this study was conducted using two seedling-stage inbred lines grown under controlled conditions, which may limit the generalizability of the results to diverse germplasm backgrounds, other tissues, developmental stages, and field environments, where abiotic and biotic stresses may influence anthocyanin accumulation. Second, the genetic divergence between the two lines precludes the unequivocal attribution of the observed metabolic and transcriptional differences solely to anthocyanin-specific regulation, as these differences may partly reflect genotype-dependent variation in growth and vigor. Third, the WGCNA-derived modules and gene–metabolite correlations are inherently correlative and do not establish causal relationships. Future studies should functionally validate the prioritized MYB hub genes, *Zm00001d018013* and *Zm00001d039417*, through gene editing combined with metabolic flux analysis. Extending this work to broader germplasm panels, multiple tissues and developmental stages, and field trials will also be essential for evaluating the agronomic relevance and translational potential of the proposed model.

## 5. Conclusions

Anthocyanins contribute to stress responses, nutritional value, and crop quality, making them important targets for crop improvement. This study integrates time-resolved metabolomic and transcriptomic analyses to characterize molecular features associated with anthocyanin accumulation in maize. By comparing anthocyanin-rich and anthocyanin-deficient lines across multiple developmental stages, we identified a refined set of metabolites and genes linked to pigmentation and revealed two antagonistic transcriptional modules with opposing associations with anthocyanin content. Integration of these datasets supports a model in which anthocyanin accumulation is associated with MYB-centered transcriptional programs that coordinate flavonoid biosynthesis alongside broader metabolic processes related to precursor supply and redox balance. The candidate genes and co-expression modules identified here provide a focused set of targets for future functional studies and offer a framework for connecting transcriptional regulation with metabolic output in developmentally regulated traits. Together, these findings advance our understanding of how anthocyanin accumulation is embedded within broader metabolic networks and provide a path for efforts to modulate pigmentation in maize. Future studies should prioritize functional validation of the candidate MYB hub genes, extension of this analytical framework to broader germplasm and field environments, and assessment of potential trade-offs between anthocyanin-associated traits and yield performance in breeding programs.

## Data Availability

The original contributions presented in this study are included in the article/Supplementary Materials. Further inquiries can be directed to the corresponding author.

## Supplementary Materials

The following supporting information can be downloaded at: https://www.mdpi.com/article/10.3390/genes17080866/s1, Table S1. Metabolite abundance data for PH19401 and YPX. Table S2. The detected metabolites were annotated using KEGG, HMDB, and LipidMaps databases. Table S3. Differential metabolites between PH19401 and YPX across different comparison groups. Table S4. KEGG enrichment analysis results of candidate core metabolites. Table S5. Transcriptome Sequencing Data Output and Quality Statistics. Table S6. List of high-confidence anthocyanin-related DEGs from the PH × YP intersection. Table S7. KEGG enrichment analysis results of candidate DEGs. Table S8. Relative abundance of 60 anthocyanin-related core metabolites across 30 samples. Table S9. Expression levels (FPKM) of the 14 candidate genes across all samples. Table S10. Pearson correlation coefficients between the expression profiles of all annotated MYB family genes and the 14 candidate genes across all samples.
